# It’s never lupus: A case of atypical psychosis and neuropsychiatric lupus

**DOI:** 10.1192/j.eurpsy.2021.2108

**Published:** 2021-08-13

**Authors:** S. Jesus, A. Costa, J. Alcafache, P. Garrido

**Affiliations:** Psiquiatria E Saúde Mental, Centro Hospitalar do Baixo Vouga, Aveiro, Portugal

**Keywords:** Systemic Lupus Erythematosus, Neuropsychiatric Lupus, psychosis

## Abstract

**Introduction:**

Systemic lupus erythematosus (SLE) is a chronic autoimmune disease involving the production of autoantibodies with consequent involvement of multiple organ systems. Although not an uncommon condition, its pleomorphic neuropsychiatric manifestations imply consideration of SLE as a relevant differential diagnosis. As many as 50% of patients with SLE have neurological involvement throughout their disease course and it is associated with impaired quality of life, high morbidity and mortality rates.

**Objectives:**

Case report study and discussion.

**Methods:**

The authors present a case of a 50-year old woman without previous psychiatric history presenting to the psychiatric department with suicidal ideation in association with psychotic symptoms of rapid onset. She presented with various somatic symptoms including butterfly rash, alopecia, nail dystrophy and generalized myalgia and arthralgia. After conducting a thorough clinical investigation with subsequent unveiling of various alterations including those in the antibody panels and abnormal magnetic resonance imaging results, a diagnosis of neuropsychiatric lupus was established.

**Results:**

Improvements in initial psychiatric symptoms were noted after completing pulse corticoid therapy for SLE with adjunct antipsychotic medication. On follow-up, the patient demonstrated a complete return to previous mental functioning with no reported relapses.

**Conclusions:**

This case demonstrates the heterogeneous presentations that neuropsychiatric lupus can assume. The vast array of psychopathological signs and symptoms in SLE continue to exist as a significant diagnostic and therapeutic challenge. Timely identification resulting from a proactive approach in maintaining lupus as part of our differentials may prevent the significant morbidity and mortality commonly associated with the resultant central nervous system involvement in SLE.

**Disclosure:**

No significant relationships.

